# Case Report: Eltrombopag in mosaic and gene therapy-treated patients with Fanconi anemia

**DOI:** 10.3389/fped.2025.1625751

**Published:** 2025-08-11

**Authors:** Josune Zubicaray, June Iriondo, Elena Sebastián, Alejandro Sanz, Paula Rio, Jean Soulier, Sonsoles San Román, José J. Uriz, Susana Navarro, Eileen Nicoletti, Juan A. Bueren, Jonathan D. Schwartz, Julián Sevilla

**Affiliations:** ^1^ Pediatric Hematology and Oncology Department and Foundation for the Biomedical Research, and Biomedical Network Research Center for Rare Diseases (CIBERER), Pediatric University Hospital Niño Jesús, Madrid, Spain; ^2^ Biomedical Innovation Unit, Center for Research on Energy, Environment and Technology (CIEMAT), Biomedical Network Research Center for Rare Diseases (CIBERER) and Sanitary Research Institute Fundación Jiménez Díaz, Madrid, Spain; ^3^ Institut de Recherche Saint-Louis, Inserm, CNRS, and Hôpital Saint-Louis, APHP, Université Paris Cité, Paris, France; ^4^ Pediatric Hematology and Oncology Department, La Paz University Hospital, Madrid, Spain; ^5^ Pediatric Hematology and Oncology Unit, Pediatrics Department, Donostia University Hospital, San Sebastián, Spain; ^6^ Rocket Pharmaceuticals, Inc., Cranbury, NJ, United States

**Keywords:** Fanconi anemia, bone marrow failure, eltrombopag, gene therapy, mosaicism

## Abstract

Fanconi anemia (FA) constitutes the most common of the inherited bone marrow failure syndromes, a group of rare heterogeneous disorders characterized by cytopenia, predisposition to hematologic and solid malignancies and diverse clinical features. Currently, the only available hematopoietic curative treatment for bone marrow failure is an allogeneic hematopoietic stem cell transplantation (HSCT), although gene therapy has demonstrated evidence of efficacy and substantially reduced toxicity. It has been demonstrated that eltrombopag stimulates trilineage hematopoiesis in aplastic anemia, and preclinical studies suggest it promotes DNA repair in FA hematopoietic stem cells (HSCs). Herein, we report the experience with eltrombopag in a patient misdiagnosed with aplastic anemia and subsequently determined to have FA mosaicism and in two FA patients who previously received gene therapy but who were infused with very low numbers of gene-corrected HSCs. Strikingly, the patient with somatic mosaicism achieved transfusion independence and averted HSCT, and the gene-therapy patients showed a marked increase of corrected cells during treatment.

## Introduction

Fanconi Anemia (FA) is a rare genetic disorder characterized by bone marrow failure (BMF), predisposition to cancer and physical abnormalities. In approximately 80% of the patients, the characteristic BMF becomes evident during the first decade of life, requiring treatment in most cases (1, 2). Although hematopoietic stem cell transplantation (HSCT) is currently the only curative therapy for FA-related BMF, recent gene therapy studies suggest that infusion of autologous gene-corrected hematopoietic stem cells (HSCs) offers a less-toxic therapeutic alternative capable of preventing or even reverting BMF (3, 4).

It is known that some FA patients are able to spontaneously revert the pathogenic mutation in hematopoietic cells, restoring their DNA repair capacity and conferring a proliferative advantage over the non-reverted cells. Several studies report that these patients show a milder course, with reduced incidence of BMF or even the ability to spontaneously improve peripheral blood (PB) cell counts, a decreased incidence of hematological malignancy and a lower mortality in the first decades of life (5, 6). Additionally, in a recent gene therapy clinical trial we have demonstrated that gene-corrected HSCs acquire a marked proliferation advantage *in vivo*, and can even reverse the BMF progression, mimicking the behavior of reverted HSCs in mosaic patients (4).

Supportive therapy primarily includes transfusion therapy and androgens, although other medications are being tested in clinical trials. Eltrombopag (EPAG), a non-peptide thrombopoietin receptor agonist, has been approved for severe aplastic anemia (SAA), based on its capacity to stimulate trilineage hematopoiesis (7, 8). Some reports have also suggested a potential efficacy of this drug in FA, although the available information is scarce. Preclinical studies have shown that stimulation of TPO/MPL signaling by eltrombopag promotes DNA repair in HSCs through the classic non-homologous end joining repair mechanism, improving genome integrity, cell survival, and HSC functionality (9, 10). In addition, a study demonstrated that eltrombopag is capable of bypassing the IFNγ-mediated inhibition in the endogenous TPO signaling pathway in HSCs *in vitro*, which could explain its efficacy in SAA (11). Though not uniform, some data indicate IFNγ is also overexpressed in FA patients along with other pro-inflammatory cytokines, suggesting an additional potential therapeutic mechanism of eltrombopag (12–16). Regarding clinical evidence, Gupta et al. published in 2018 the first case in which the combination of EPAG with oxymetholone stimulated significant trilineage hematopoiesis in a patient, eliminating transfusion requirements (17). Later, Koker et al. published a similar case in a 5-year-old male who achieved platelet transfusion independence after adding EPAG to baseline oxymetholone treatment (18). In both cases, EPAG was used as a bridge to HSCT and patients were subsequently transplanted. Moreover, Barranta et al. reported preliminary results of the first 10 patients enrolled in an ongoing clinical trial that explores the use of eltrombopag in FA (NCT03206086). At 6 months, 2 of the 4 evaluable patients showed a response in PB (platelet increase >20 × 10^9^/L above baseline, Hb >15 g/L, or ANC >0.5 × 10^9^/L), and all four achieved a bone marrow response, defined as a minimum of two-fold increase in the mean marrow cellularity or in the proportion of CD34^+^ cells (19).

Regarding the use of EPAG in other IBMFS, there is some limited evidence in Diamond-Blackfan Anemia (DBA) and dyskeratosis congenita (DC). In DBA, the exact mechanism by which erythropoiesis is altered is unclear, but according to one theory, an imbalance between heme and globin synthesis would lead to the accumulation of free heme in the cell, inducing cell death through apoptosis and ferroptosis. In a preclinical model exploring the effect of eltrombopag on erythropoiesis in induced pluripotent stem cells (iPSCs) from patients with DBA, the incorporation of eltrombopag in the early stages of differentiation improved erythropoiesis through the chelation of intracellular iron (20). This, together with some isolated experience of hematologic response in one patient (21), led to the development of a phase I/II clinical trial at the NIH (NCT04269889), which included 15 patients with ABD refractory or intolerant to corticosteroids. Only one of the 15 patients achieved a hematologic response, going from receiving 1.8 transfusions every 8 weeks to receiving none, and with a hemoglobin increase up to 124 g/L at 6 months of treatment (22).

In the case of DC, we could only find 2 case reports reporting the use of EPAG in 3 patients, without any hematologic improvement (although authors used lower doses of the drug, similar to the ones used in immune thrombocytopenia) (23, 24). Furthermore, the drug has not been shown to have any effect on telomere length in a study in SAA (25).

To our knowledge, this drug has not been specifically investigated in mosaic or gene therapy-treated populations in FA. Herein, we report for the first time the impact of EPAG in an FA patient with mosaicism and two additional FA patients who received lentiviral mediated gene therapy in the FANCOLEN-I trial (NCT03157804) and had been infused with a very low dose of transduced CD34^+^ cells.

## Methods

Biological and clinical information from the mosaic patient before the referral to our center was provided by the hospital of origin. The diepoxybutane (DEB) chromosomal fragility test was performed on PB T-lymphocytes, and the mitomycin C (MMC) resistance in bone marrow (BM) was assessed by clonogenic assays, as previously described (4, 5). Sanger sequencing was used for confirmation of the reversal of the pathogenic mutation in BM cells from the mosaic. Cytogenetic aberrations in BM were studied by karyotype analysis by G-banding and by FISH [CDKN2C/CKS1B (1p32/1q21), RPN1/MECOM (3q21.3/3q26.2) and Vysis D7S486/CEP7]. Furthermore, in the patients previously treated by gene therapy, Agilent 400 K Array CGH technology was also used in genomic BM DNA, as well as a custom gene panel with genes related to myeloid neoplasms was used for targeted gene-sequencing on a MiSeq system (Illumina) (4). Analysis of the lentiviral vector copy number (VCN) in patients that had received gene therapy was performed as previously described by our group (4, 26). We defined hematologic response according to the criteria used in our clinical trial that explores the use of eltrombopag in FA (NCT06045052), as follows: Complete response (CR) was defined as a platelet count >50 × 10^9^/L, Hb >100 g/L and ANC >1 × 10^9^/L without transfusion requirements in the 4 weeks prior to evaluation. Partial response (PR) was defined as improvement in at least one hematopoietic lineage, as follows: platelets >50 × 10^9^/L, hemoglobin >100 g/L or ANC >1 × 10^9^/L without transfusion requirements in the 4 weeks prior to evaluation.

## Case description and discussion

**Figure 1:**
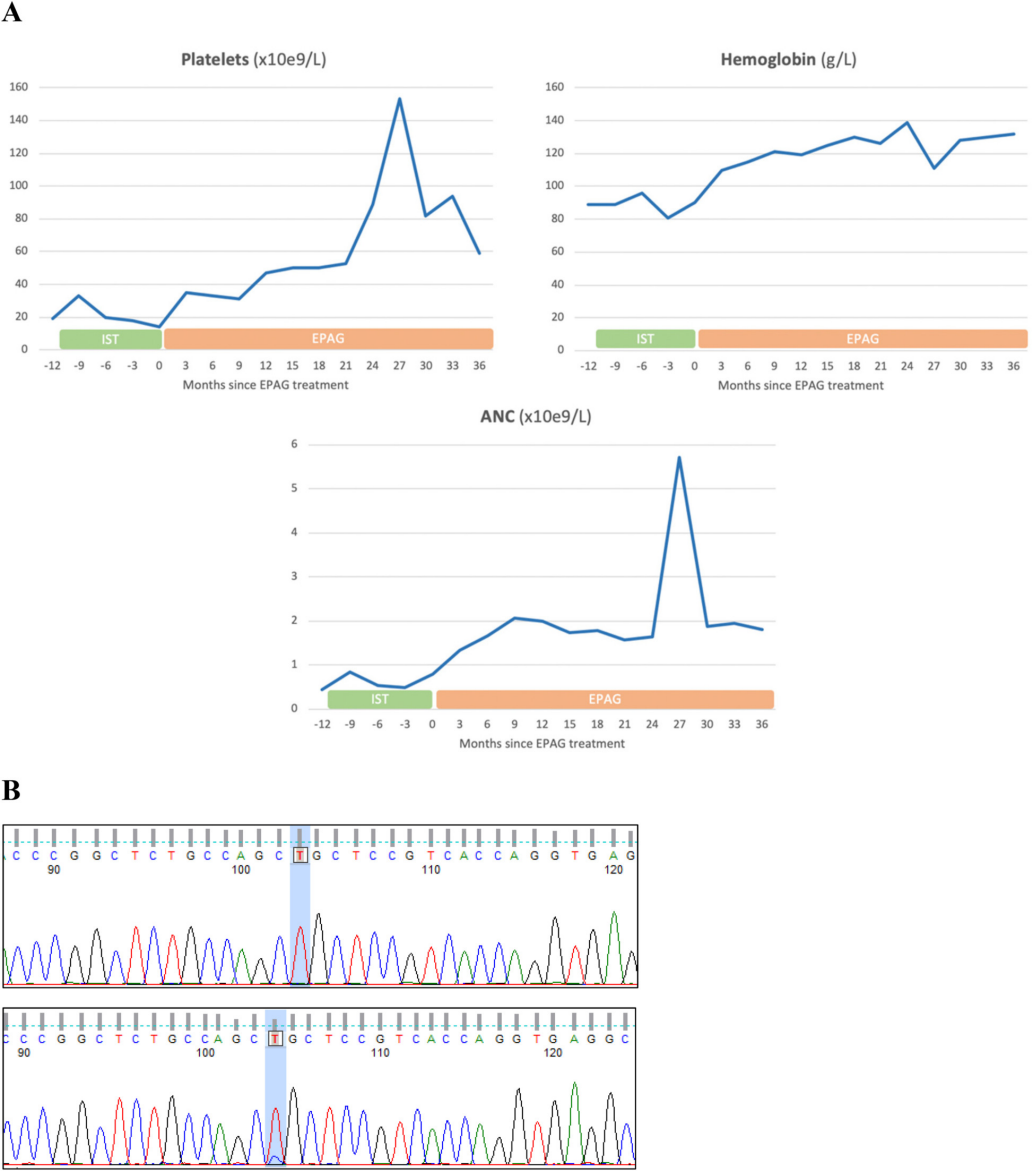
Clinical and molecular evolution of the Fanconi anemia patient who received eltrombopag due to a misdiagnosis of severe aplastic anemia. **(A)** Evolution of peripheral blood counts before and during eltrombopag treatment. **(B)** Molecular study of the *FANCA* gene demonstrating reversion of one of the pathogenic mutations [c.2303T>C p.L768P (exon 25)], performed at the time of FA diagnosis. The graph reflects in the top box the study carried out on MMC-resistant colonies and therefore fully reverted (T 100%), and in the bottom box the study carried out on the whole bone marrow aspirate sample where it is shown in the same position how some of cells still carry the mutation and do not code for T. IST, immunosupressive therapy; EPAG, eltrombopag; ANC, absolute neutrophil count.

A 7-year-old white male was initially diagnosed with SAA due to isolated severe pancytopenia and a hypocellular marrow without morphological or clonal alterations. The patient did not present any physical abnormalities, laboratory alterations nor family history suggestive of a potential congenital cause. No additional diagnostic testing was performed at the time. Due to the absence of an available matched donor for HSCT, the patient received a first course of immunosuppressive treatment (IST) with rabbit anti-thymocyte globulin (ATG) and cyclosporine A, with no response. A second cycle of IST was then administered with horse ATG and cyclosporine ten months later. After one month, due to the absence of hematologic improvement, a daily dose of 50 mg of EPAG was introduced, which was progressively increased up to 100 mg/day. As shown in Figure 1, panel A, PB counts evolved favorably in the following months. No drug-related adverse events, including clonal evolution in BM by karyotype analysis and FISH, were observed during treatment. Due to persistent mild thrombocytopenia, a diagnostic reevaluation was performed by Next Generation Sequencing (NGS) with a panel of 147 IBMFS-related genes in peripheral blood at the age of 10, three years and nine months after the initial SAA diagnosis (27). As a result, the patient was diagnosed with FA due to the presence of 2 pathogenic variants in the *FANCA* gene [c.2303T>C p.L768P (exon 25), and c.1115_1118delTTGG p.V372fs (exon 13)]. Diagnosis was confirmed by a chromosomal fragility test compatible with FA in peripheral blood lymphocytes. Then, a new BM study was performed, and, strikingy, approximately 75% of bone marrow colony forming cells (CFCs) were resistant to MMC, suggesting the reversion of the mutation in the HSCs of the patient. Somatic mosaicism was also confirmed by molecular studies in a paraffin-embedded BM sample obtained prior to the administration of EPAG at the age of 7 (Figure 1, panel B), indicating that a small reverted clone with a reversion in the exon 25 variant already existed when the patient began treatment. Importantly, at the time of FA diagnosis, PB counts had improved sufficiently to keep him transfusion and HSCT free, achieving a complete hematologic response according to our criteria described above. Given the absence of data regarding safety of EPAG in this population at that time, treatment was discontinued and substituted with danazol, which was discontinued years later due to full recovery. At the time of last follow up in September 2024, the patient remains hematologically stable with hemoglobin 150 g/L, platelets 115 × 10^9^/L, and neutrophils 1.4 × 10^9^/L, and without requirement for any supportive care for BMF.

Regarding the two patients previously treated with gene therapy, we have recently reported the hematologic and molecular response of FA patients included in the phase I/II gene therapy clinical trial FANCOLEN-I (NCT03157804) (28). Most patients infused with very low numbers of corrected CD34^+^ cells (less than 240,000 corrected CD34^+^ cells/kg), experienced BMF progression and subsequently became transfusion-dependent (4, 28). Two of those patients received EPAG off-label over 6 and 11 months, respectively. The first of them (patient 2) corresponds to a 5-year-old arab boy at the time of EPAG initiation, who was diagnosed with FA at one year of age due to alterations in the *FANCA* gene (Del EX 1–43; C. 3788_3790 Del TCT). The patient presented an ectopic kidney, hypoplasia of both thumbs and a permeable ductus arteriosus, fulfilling the VACTER-L association criteria (at least 3 of the following: Vertebral abnormalities, Anal atresia, Cardiac abnormalities, Tracheo-esophageal fistula, Esophageal or duodenal atresia, Renal abnormalities, upper Limb abnormalities and Hydrocephalus), and also presenting with 2 of the PHENOS features (low-set ears and short stature out of skin Pigmentation abnormalities, small Head, small Eyes, structural central Nervous system abnormalities, Otologic abnormalities and Short stature) (29). He developed severe BMF by the age of 3, which prompted his participation in the mentioned gene therapy trial, and was infused with 158,400 autologous corrected CD34^+^ cells/kg. One year and 7 months after infusion, due to the worsening of PB cell counts, the patient started treatment with 2.5 mg/Kg/day of EPAG off-label (50 mg/day for 4 days and 25 mg/day for 3 days a week because tablets were the only available formulation at the time), which he received for 6 months. The other patient (patient 3), a 10-year-old white boy at the time of EPAG initiation, was diagnosed with FA at the age of 5 years and fulfilled the same VACTER-L criteria as patient 2, associating only short stature of the PHENOS features. He also presented with alterations of the *FANCA* gene (C. 1,115–1,118 Del TTGG; C. 1115–1118 Del TTGG). He developed severe BMF by the age of 7, and was infused with 163,030 autologous corrected CD34^+^ cells/kg. Three years and 1 month after infusion, due to the worsening of PB cell counts, the patient started treatment with EPAG at a dose of 75 mg/day, which he received for 11 months. It is worth to mention that the EPAG dose of these patients was based on that used in the clinical trial of EPAG and IST in SAA by Townsley et al. (8) No medication-related adverse events, including clonal evolution (assessed by karyotype analysis, FISH, array CGH and a somatic variant analysis by a myeloid neoplasm-related gene panel), were observed during the follow-up of these patients.

**Figure 2:**
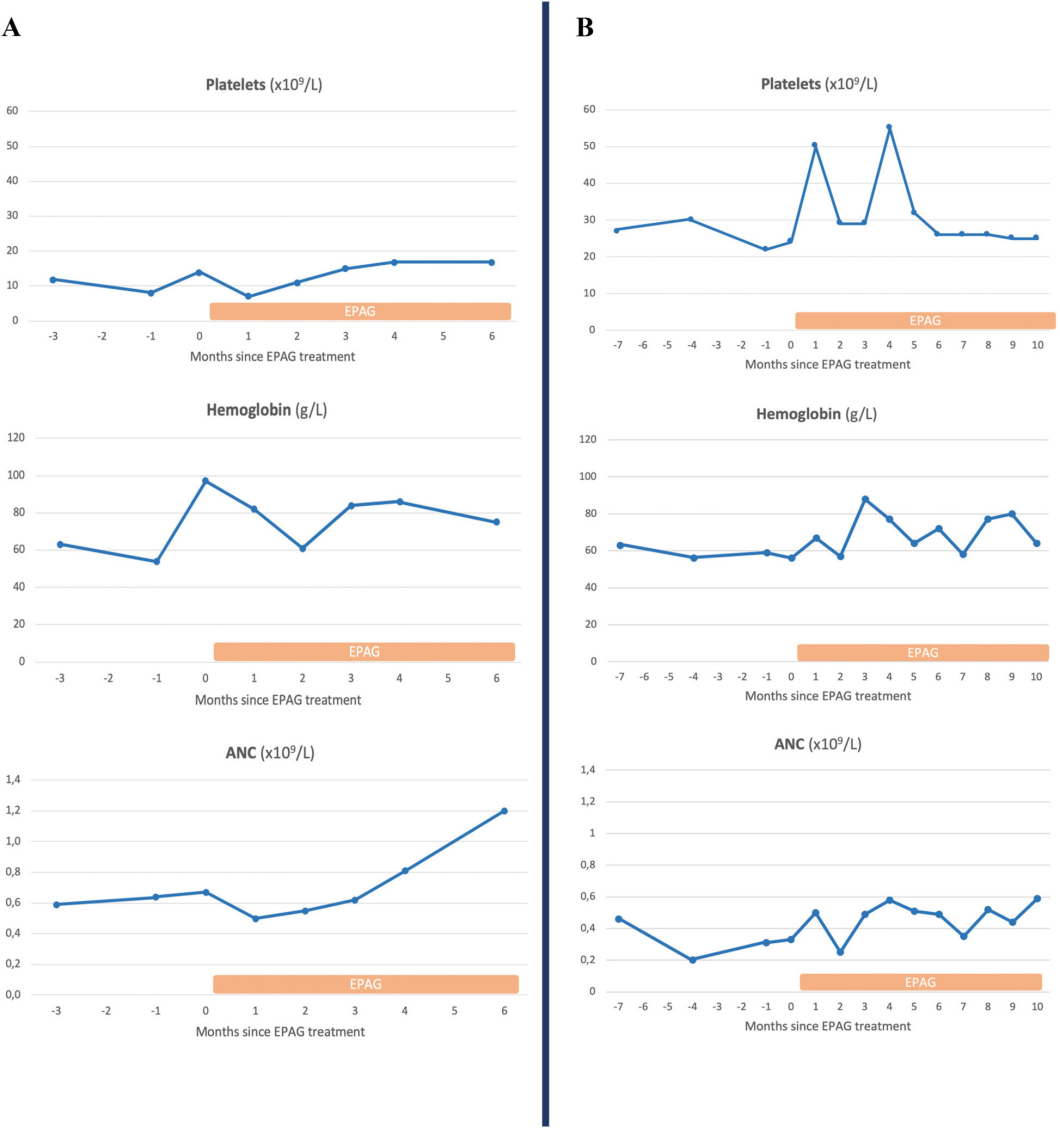
Evolution of peripheral blood cell counts in the two patients previously treated by gene therapy before and during treatment with eltrombopag. **(A)** patient 2. **(B)** patient 3. EPAG, eltrombopag; ANC, absolute neutrophil count.

**Figure 3:**
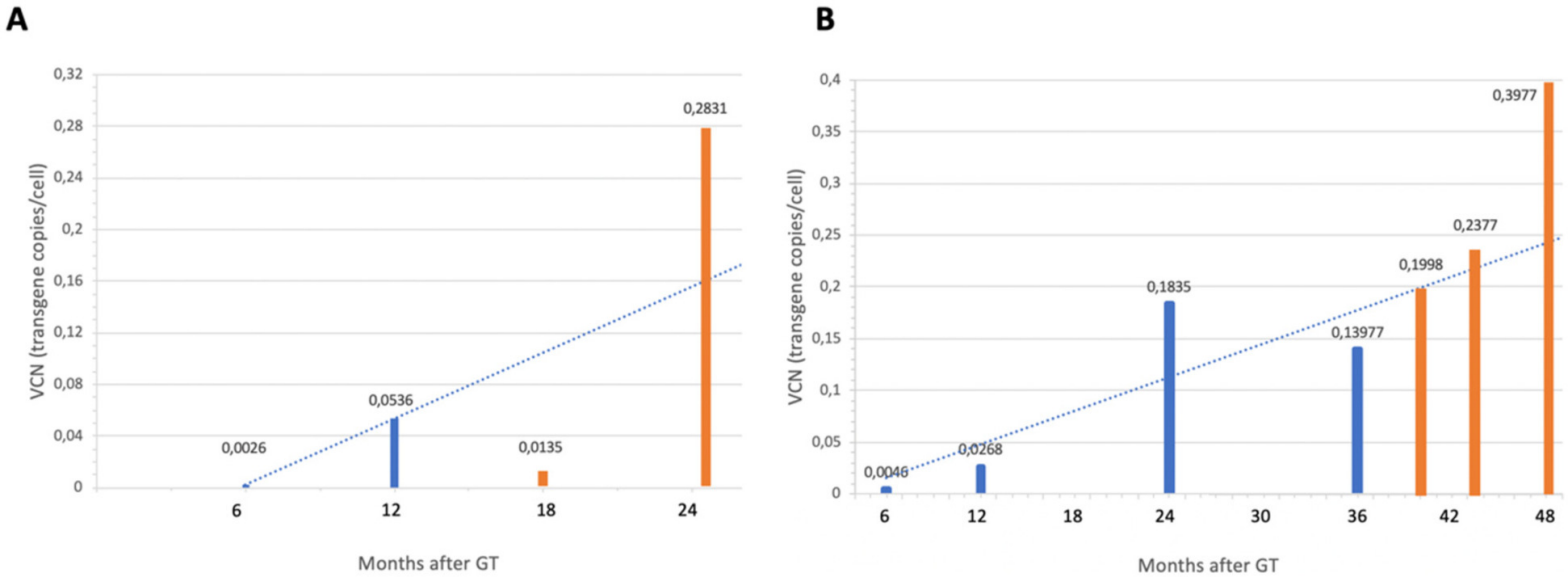
Vector copy number (VCN) evolution in bone marrow (BM) samples of the two gene therapy (GT)-treated patients before and after eltrombopag. Samples before eltrombopag (in blue) were obtained every 6 months since the infusion date. After initiation of eltrombopag (in orange), samples were obtained at 3, 6 and 12 months. **(A)** Patient 2. **(B)** Patient 3.

Unfortunately, no significant hematologic response was observed in PB cell counts after treatment with EPAG, and both patients had to proceed to HSCT given the severity of the BMF (Figure 2). However, surprisingly, a greater-than-expected increase in the proportion of corrected cells was observed as compared to the trajectories prior to EPAG (Figure 3). In evaluable BM samples, increases in VCN were associated with an increase in the proportion of MMC-resistant CFCs, revealing the phenotypic correction of gene-corrected HSCs (Table 1).

**Table 1:** Evolution of cellularity, frequency of CD34^+^ cells and the proportion of MMC-resistant colony forming cells in bone marrow samples of the two gene-therapy patients before and after eltrombopag.

Patient 2	Pre-EPAG				Post-EPAG		
Time since GT (months)	6	12	25		28	31	
Celullarity (×10^6^ cells/mL)	8.8	5.3	4.46		4.06	6.11	
CD34+ cells (%)	0.2	0.104	0.13		0.095	0.024	
CFCs/100,000 cells	NP	NP	17.5		11.1	8.9	
MMC (10nM) resistant CFCs (%)	NP	NP	8.4		27.7	28.4	
Patient 3	Pre-EPAG				Post-EPAG		
Time since GT (months)	6	12	24	36	40	43	48
Celullarity (×10^6^ cells/mL)	6.7	5.9	6.3	4.5	2.5	4.25	3
CD34+ cells (%)	0.28	0.198	0.043	0.049	0.057	0.078	0.012
CFCs/100,000 cells	11.87	7.2	8.67	0.93	5.6	9.9	4.1
MMC (10nM) resistant CFCs (%)	1.9	7.4	16.9	NP	21.4	22.4	NP

EPAG, eltrombopag; GT, gene therapy; CFCs, colony forming cells; MMC, mitomicin C; NP, not performed.

Altogether, in this report we present novel findings related to the use of EPAG in FA patients, particularly the subgroup of patients with mosaicism (either naturally occurring somatic mosaicism as in the first case, or “artificial mosaics” created by gene-therapy as patients 2 and 3), since we report for the first time an enhancement of the expansion of reverted or gene-corrected cells potentially favored by a drug.

Importantly, the patient with somatic mosaicism, who did not have any available donor for HSCT and remained transfusion dependent for almost a year with a severe BMF, recovered PB cell counts to the point of no longer needing a HSCT. In the case of the gene therapy patients who had been infused with very low doses of corrected CD34^+^ cells, we observed a substantial increase in the proportion of gene-corrected cells. We hypothesize the lack of significant hematological response might be due to the late introduction and short duration of EPAG treatment (patients were unlikely to have sufficient corrected cells to restore hematopoiesis), to the moderate dose of the drug, and/or the poor hematopoietic reservoir of these patients.

As mentioned before, some preclinical studies have proposed different mechanisms by which EPAG could exert a beneficial effect on FA HSCs (9, 11). However, although the main mechanism by which HSCs disappear in FA is thought to be the cumulative DNA damage in the cell, several other mechanisms have been described and etiology is thought to be multifactorial (14). Especially in advanced stages of BMF, the HSC reservoir is severely diminished, which reduces the capacity of those cells to restore hematopoiesis. Therefore, we hypothesize gene-corrected cells are probably more “sensitive” to the beneficial effects of the drug as they are less damaged, and that it is likely that their presence such as in “natural or artifial” mosaics facilitates a better response to the drug, although a sufficient quantity and time will still be necessary to be able to increase the HSC pool enough to see an improvement in PB cell counts. Our group is currently working on projects to try to answer these questions.

In fact, regarding the use of eltrombopag in other IBMFS, investigators of the clinical trial in DBA mentioned above report that the index patient who motivated the development of the trial, a patient with mutation in the *RPL11* gene who improved dramatically after 16 weeks of treatment with EPAG and showed recurrent anemia after discontinuation of treatment, was a mosaic by uniparental disomy at the chromosome 19 locus, with a majority of healthy HSC compared to mutated HSC. This, together with their experiments in murine models, suggests that the mutated HSC may inhibit the expansion and differentiation of healthy cells by interfering with the macrophage support function of erythroblast islets, and that the index patient needed less drug stimulation because he had more healthy cells (22, 30). Further studies are required to determine whether a similar phenomenon could be occurring in FA.

We believe that the presented data suggest the potential benefit of an adjuvant treatment with EPAG in those patients infused with low numbers of corrected cells in order to boost the repopulation of corrected cells.

Remarkably, we did not observe any significant adverse event nor clonal evolution in these patients, although we acknowledge the treatment period in the gene-therapy treated patients was relatively short.

Our findings on the presented patients prompted the design of the FANCREV clinical trial (NCT06045052), which aimed to explore the safety and efficacy of EPAG in FA patients (including patients with somatic mosaicisim and previous gene therapy). The trial has recently been concluded and has been submitted for publication. Preliminary results indicated that this greater-than-expected increase in the proportion of corrected cells is confirmed in a patient also infused with very few corrected CD34^+^ cells/kg (31–33).

Taken together, this work shows for the first time a marked response to EPAG of naturally-reverted and gene therapy-corrected HSCs in FA. These results demonstrate the use of EPAG as a potential adjuvant therapy to promote the expansion of corrected cells in FA mosaic patients and those treated with gene therapy with less optimal cell doses with the goal of hematologic stabilization. Evaluation of EPAG in these patient populations is warranted in subsequent trials.

## Data Availability

The raw data supporting the conclusions of this article will be made available by the authors, without undue reservation.
